# Comprehensive Clustering Analysis and Profiling of COVID-19 Vaccine Hesitancy and Related Factors across U.S. Counties: Insights for Future Pandemic Responses

**DOI:** 10.3390/healthcare12151458

**Published:** 2024-07-23

**Authors:** Morteza Maleki, SeyedAli Ghahari

**Affiliations:** 1 School of Industrial and Systems Engineering, Georgia Institute of Technology, Atlanta, GA 30332, USA; 2 Department of Civil and Environmental Engineering, Purdue University, West Lafayette, IN 47907, USA; sghahari@purdue.edu

**Keywords:** COVID-19, vaccination, cluster analysis, public health, geospatial analysis

## Abstract

This study employs comprehensive clustering analysis to examine COVID-19 vaccine hesitancy and related socio-demographic factors across U.S. counties, using the collected and curated data from Johns Hopkins University. Utilizing K-Means and hierarchical clustering, we identify five distinct clusters characterized by varying levels of vaccine hesitancy, MMR vaccination coverage, population demographics, and political affiliations. Principal Component Analysis (PCA) was conducted to reduce dimensionality, and key variables were selected based on their contribution to cumulative explained variance. Our analysis reveals significant geographic and demographic patterns in vaccine hesitancy, providing valuable insights for public health strategies and future pandemic responses. Geospatial analysis highlights the distribution of clusters across the United States, indicating areas with high and low vaccine hesitancy. In addition, multiple regression analyses within each cluster identify key predictors of vaccine hesitancy in corresponding U.S. county clusters, emphasizing the importance of socio-economic and demographic factors. The findings underscore the need for targeted public health interventions and tailored communication strategies to address vaccine hesitancy across the United States and, potentially, across the globe.

## 1. Introduction

The COVID-19 pandemic has indelibly marked global public health, economic stability, and societal norms. As of 2024, the virus has prompted varied responses from different jurisdictions, influenced by geographic, demographic, and socio-economic factors [1,2]. Initial measures in the United States focused predominantly on curtailing transmission through widespread lockdowns and mask mandates, often without consideration for regional disparities in healthcare access, political alignment, and public compliance levels. COVID-19 has had devastating impacts on the United States (U.S.) population. Specifically, the pandemic impacted certain populations differently than others based on demographics [3,4,5,6], financial status [3,7], behavioral/psychographics [4,8,9,10,11], and geographies [6,8]. Additionally, socio-economic factors such as exposure to media and political party affiliation [12] played an important role in influencing vaccine uptake, [9] and different jurisdictions (e.g., counties, states, and federal) had varying responses on how to control and reduce risks of the pandemic (e.g., mask mandates and school closures) [8,13].

With the introduction of multiple vaccine boosters and new vaccine technologies, understanding the current landscape of vaccination uptake is critical. This variability in pandemic responses and their outcomes presents a critical opportunity for analysis. Understanding the effectiveness of different strategies across diverse contexts is vital for preparing more resilient public health responses in the future. Thus, our study focuses on a data-driven approach to dissect these varied responses within the United States at the county level.

As the global community continues to navigate the challenges of COVID-19 and future pandemics, the insights derived from this analysis aim to contribute to a more informed, agile, and region-specific response strategy that can be adapted to the unique needs of diverse populations.

Employing clustering analysis, this research identifies patterns and correlations between pandemic outcomes and the socio-economic characteristics of counties. By applying unsupervised machine learning techniques, specifically K-Means and hierarchical clustering, we categorized counties into distinct groups based on their performance in managing COVID-19—assessed through metrics such as mortality rates and vaccine uptake. The analysis was further enhanced by Principal Component Analysis (PCA) to identify the most significant variables contributing to these outcomes, ensuring a robust dimensionality reduction and feature selection process. Specifically, we aimed to achieve the following:

1. Analyze vaccination rates and COVID-19 mortality rates at the county level.
2. Explore the relationship between these rates and various socio-economic variables such as median household income, education levels, racial composition, and political affiliation.
3. Identify distinct clusters of counties that share similar socio-economic profiles and vaccination responses.
4. Provide insights and recommendations for public health agencies to improve vaccination uptake and manage pandemic responses more effectively.

The clustering of the demographic data allowed us to identify distinct groups of U.S. counties that share similar socio-economic and demographic profiles. By doing so, we can understand how different socio-economic factors influence COVID-19 vaccine hesitancy across various regions. This approach helps in recognizing patterns and disparities that may not be evident when analyzing data on an individual basis. In addition, the criteria for selecting variables were based on their relevance to vaccine hesitancy and their availability at the county level. Key variables included socio-economic factors (e.g., median household income and higher education levels), demographic factors (e.g., racial composition and age distribution), health-related factors (e.g., vaccine hesitancy rates and COVID-19 case rates), and political affiliation (e.g., percentage of Republican voters). These variables were chosen because they are known to influence health behaviors and vaccination uptake, as documented in previous research. The selected variables provided a comprehensive overview of the factors that potentially affect vaccine hesitancy. By including socio-economic, demographic, health-related, and political factors, we aimed to capture the multi-faceted nature of vaccine hesitancy. Some variables were excluded due to a lack of reliable data at the county level or because they were not directly relevant to the focus of our study. For example, variables like federal education investment were excluded due to high levels of missing data, and variables like ICU bed occupancy were excluded because they were not directly linked to vaccine hesitancy.

This study is unique in its comprehensive approach to clustering U.S. counties based on a wide range of socio-economic, demographic, health-related, and political variables. While previous studies have focused on individual factors influencing vaccine hesitancy, our study integrated multiple variables to provide a more holistic view. Additionally, the use of advanced clustering techniques like K-Means and hierarchical clustering sets our study apart by identifying distinct county clusters that share similar profiles.

In the following section, we highlight the relevant academic research conducted on this subject to date and their implication for informing future pandemic decision making.

## 2. Literature Review

During the COVID-19 pandemic peak years of 2020–2021, the United States followed a broad, conservative approach to contain the pandemic that focused strongly on preventing transmission [8]. This was logical, given the contagious nature of the virus; however, there might have been underlying systemic differences putting certain groups of society at a bigger disadvantage than others [6,13]. Vaccine hesitancy, even to date, has been cited as a barrier to the effective control of COVID-19, therefore, it is imperative to understand its root cause to better handle future unforeseen circumstances. Previous geography-specific analyses on the spread of the pandemic were mostly limited to the number of “cases”, “deaths”, and “vaccines” [14,15,16,17]. This was due to the unavailability of the data [18] and the limitations of those data that do exist [6,19,20]. While current approaches are informative in nature, they lack the prescriptive aspect of data analysis and fail to provide recommendations on what actions should be taken to promote vaccination.

Clustering analysis has been increasingly used in recent studies to explore various dimensions of the COVID-19 pandemic. For instance, Otto et al. utilized advanced clustering techniques to examine mobility data and its correlation with virus transmission rates across Europe, highlighting how mobility patterns could predict outbreak severity [21]. The integration of socio-economic data into clustering analyses has also been a critical development, revealing disparities in health outcomes. A study by Paul et al. combined health data with economic indicators using a multi-layered clustering approach, identifying high-risk areas and suggesting targeted interventions [22].

In the United States, studies have applied K-Means and hierarchical clustering to better understand the socio-economic drivers behind the pandemic’s impact. For example, Callaghan et al. and Fridman et al. demonstrated that political beliefs and party affiliations significantly impact individuals’ willingness to get vaccinated against COVID-19. Areas with higher proportions of Republican voters tend to have lower vaccination rates, likely due to differing attitudes towards the pandemic and vaccination policies [23,24].

Clustering techniques have also been used to analyze spatial and temporal patterns of disease spread, healthcare access, and vaccination behaviors. Singh et al. utilized hierarchical clustering to identify regions with similar healthcare access and outcomes, providing insights for targeted healthcare interventions [25]. Wang et al. used K-Means clustering to categorize countries based on their COVID-19 response strategies, revealing distinct clusters with varying levels of success in controlling the virus [26].

Combining socio-economic variables with vaccination data in clustering analysis provides a comprehensive view of the factors influencing vaccine uptake. Biswas et al. conducted a clustering analysis integrating socio-economic, demographic, and health-related variables to study the determinants of COVID-19 vaccine acceptance in India, highlighting the importance of considering multiple dimensions to understand vaccine hesitancy comprehensively [27]. Similarly, Barber and Kim integrated socio-economic and behavioral data in their clustering analysis of influenza vaccination rates, identifying distinct clusters with unique vaccination behaviors and socio-economic profiles [28]. These studies underscore the utility of clustering analysis in understanding and managing pandemics by linking data-driven insights with practical policy applications. They also highlight the potential for these techniques to facilitate better preparedness and response strategies for future global health crises.

While significant progress has been made in understanding the determinants of COVID-19 vaccination uptake, gaps remain in the literature. Many studies have focused on individual factors, such as income, education, or political affiliation, without considering the combined impact of these variables. Additionally, there is limited research on the application of advanced clustering techniques to analyze vaccination behaviors at the county level in the United States. This study aims to fill these gaps by employing hierarchical and K-Means clustering to analyze the combined impact of multiple socio-economic variables on COVID-19 vaccination rates at the U.S. county level. Additionally, we aim to employ multiple regression analysis within each formed cluster to evaluate the impact of various socio-economic variables on vaccine hesitancy for the corresponding cluster of counties to provide insights on how targeted policy making can assist in tackling issues in the times of pandemic. In the following sections, the data sources and methodologies to accomplish this goal are provided in detail.

## 3. Materials and Methods

This section outlines the structured approach undertaken to gather, clean, analyze, and visualize the data used in our clustering analysis of COVID-19 responses at the county level in the United States. The methodology is divided into two main areas: (1) data collection and cleaning and (2) computation and analysis.

### 3.1. Data Collection and Cleaning

The data utilized for this study were obtained from a study by Dong et al. [29] at the Johns Hopkins University where various healthcare-related and socio-economic variables were selected to ensure a robust and comprehensive dataset. The data were sourced from various reputable agencies including the Economic Research Service (ERS) at the United States Department of Agriculture (USDA) [30], the Census Bureau [31], Johns Hopkins University (JHU)’s COVID-19 research center [32], and Harvard University’s dataverse [33]. Table 1 summarizes variables used in this study and, to ensure uniformity, variables were codified into concise labels as summarized. The codification of each variable follows the XX-YY pattern, where XX is a two-letter abbreviation for the category of the variable and YY is a two-letter abbreviation for the name of the variable itself. For instance, the median household income was labeled as SO-MI, SO representing *socio-economic* category and MI representing *median household income*.

The obtained data account for 3063 counties (from the total of 3142 counties) in the United States. There were a total of 79 counties not included in this study due to the unavailability of some data among the variables used in this study. The missing counties are mostly located in Nebraska, South Dakota, and Iowa in the United States.

It is important to note that vaccine hesitancy (HE-VH) data were obtained from the work by Tiu et al. [34] who leverage the COVID-19 vaccination tracking dashboard maintained by Georgetown University [35]. The vaccine hesitancy metric in the collected data was based on the estimates provided by the U.S. Department of Health & Human Services [36] by means of surveying the population within the United States. Additionally, COVID-19 case rates (HE-CD, HE-CS) refer to cumulative death cases due to COVID-19 per 100,000 people as of December 2021 and September 2021, respectively. The reason for inclusion of the data for both timeframes can be attributed to COVID-19 vaccine approval in November 2021 in the United States; therefore, a measure of the COVID-19 rate before and after vaccine approval was provided for comparison purposes. However, due to high correlation, only one of the COVID-19 case rate variables (HE-CD) was used as a predicting variable in the regression analysis to avoid multicollinearity.

Finally, a preliminary analysis of the distribution of the variables used in this study was performed by means of density plots to better comprehend the variations compared to one another. Density plots in Appendix A.1 illustrate this notion further. It is important to note that these figures have been augmented with the cluster categories obtained from the K-Means model to better assist with the comparison.

Subsequently, we screened the data for completeness and accuracy, removing any variables with more than 50% missing values to maintain the quality of our analysis. Non-numeric and irrelevant columns were excluded, and missing values were addressed by removing rows with incomplete data. These data cleaning and pre-processing steps provided a solid foundation for the subsequent clustering and analytical procedures.

**Figure 1:**
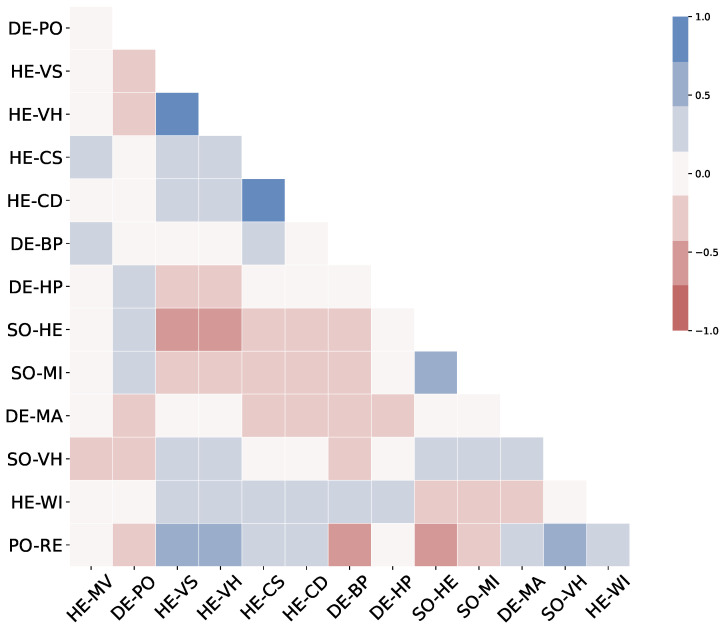
Correlation matrix of socio-economic, demographic, and health-related variables. Positive correlations are indicated by shades of blue, while negative correlations are indicated by shades of red. The strength of the correlation is represented by the color intensity. **Variable labels: DE-PO**: population 2020, **HE-VS**: vaccine hesitancy September 2021, **HE-VH**: vaccine hesitancy December 2021, **HE-CS**: case rate September 2021 (cumulative COVID-19 death cases per 100,000 people), **HE-CD**: case rate December 2021 (cumulative COVID-19 death cases per 100,000 people), **DE-BP**: Black population (%), **DE-HP**: Hispanic population (%), **SO-HE**: higher education (%), **SO-MI**: median household income, **DE-MA**: median age, **SO-VH**: vehicles per household, **HE-WI**: without insurance (%), **PO-RE**: Republican (%), **HE-MV**: measles, mumps, and rubella (MMR) vaccination coverage (0–1).

Next, we examined the correlation matrix to understand the relationships between different variables. The correlation plot in Figure 1 revealed several notable associations. For instance, vaccine hesitancy rates in December 2021 (HE-VH) were negatively correlated with the percentage of individuals with higher education (SO-HE). Conversely, political affiliation (percentage of Republican voters (PO-RE)) showed a positive correlation with vaccine hesitancy (HE-VH), aligning with previous findings that political beliefs significantly impact vaccination behaviors.

In addition to the preliminary and exploratory analyses of the data, various computational methods were employed to better understand and interpret the relationship among variables and profiles of the population. Table 2 summarizes these methodologies and the applicable uses/domains they were applied to in this work. More in-depth explanations and applications are provided in the subsequent sections.

**Table 1:** Description of variables used in the analysis.

Code	Category	Variable Name	Description
**DE-FI**	Demographic	FIPS	County-level FIPS code [30]
**DE-PO**	Demographic	Population 2020	Population as of 2020 [31]
**DE-MA**	Demographic	Median Age	Median age of the population [37]
**DE-BP**	Demographic	Black Population (%)	% of the population that is Black alone [38]
**DE-HP**	Demographic	Hispanic Population (%)	% of the population that is Hispanic or Latino [39]
**SO-HE**	Socio-economic	Higher Education (%)	% of adults with a high school diploma only, 2015–2019 [30]
**SO-MI**	Socio-economic	Median Household Income	Median household income [40]
**SO-VH**	Socio-economic	Vehicles per Household	Average number of vehicles per household [41]
**HE-MV**	Health	MMR Vaccination Coverage	Measles, mumps, and rubella (MMR) vaccination coverage (0–1) [42]
**HE-VH**	Health	Vaccine Hesitancy December 2021	% of the population hesitant to vaccinate as of 15 December 2021 [35,36]
**HE-VS**	Health	Vaccine Hesitancy September 2021	% of the population hesitant to vaccinate as of September 2021 [35]
**HE-CD**	Health	Case Rate December 2021	Cumulative COVID-19 death cases per 100,000 people as of December 2021 [35]
**HE-CS**	Health	Case Rate September 2021	Cumulative COVID-19 death cases per 100,000 people as of September 2021 [35]
**HE-WI**	Health	Without Insurance (%)	% of people who reported not having health insurance [43]
**PO-RE**	Political	Republican (%)	% of Republican voters during the 2020 presidential election [44]

These initial findings provided an overview of the socio-economic factors influencing vaccination rates and highlighted the complex interplay between these variables. The insights gained from the correlation analysis provided insights for a more detailed clustering analysis, enabling us to categorize counties into distinct groups with similar characteristics and vaccination responses.

In the following sections, additional statistical methods are employed to set the stage for a more comprehensive clustering analysis.

### 3.2. Computation and Analysis

**Figure 2:**
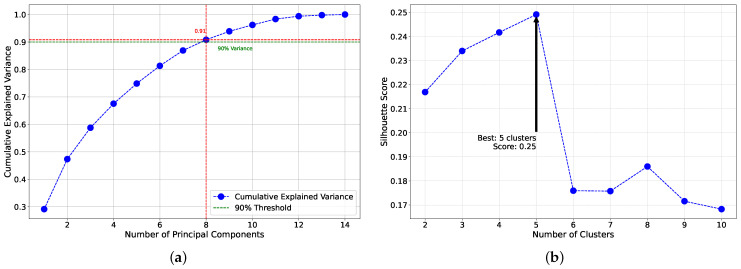
Evaluation of principal components and optimal number of clusters. This figure consists of two subplots: (**a**) cumulative explained variance by principal components. This plot shows the cumulative explained variance as a function of the number of principal components. The point where the curve starts to plateau indicates the optimal number of components to retain for capturing the majority of the variance in the dataset. (**b**) Silhouette scores for different numbers of clusters. This plot evaluates the silhouette scores for different numbers of clusters (k) in the K-Means clustering algorithm. The optimal number of clusters is indicated by the peak silhouette score, suggesting the best cluster separation. These analyses guide the selection of the number of principal components and the optimal number of clusters for the K-Means clustering algorithm.

To ensure a robust clustering analysis, we scaled the data to normalize the variables. This step was crucial for mitigating the impact of different units and scales, ensuring that each variable contributed equally to the clustering process. Next, we performed Principal Component Analysis (PCA) to reduce the dimensionality of the dataset. The cumulative explained variance plot (Figure 2a) was used to determine the optimal number of principal components. This plot illustrates the cumulative proportion of variance explained by each additional principal component. We observed that the first eight principal components accounted for 90% of the total variance in the data, which informed our decision to retain these eight components for subsequent clustering analysis.

To identify the optimal number of clusters, we employed the silhouette method. The silhouette plot (Figure 2b) evaluates the separation distance between the resulting clusters, helping to determine the number of clusters that best fit the data. The silhouette analysis indicated that five clusters provided the best separation, as evidenced by the highest silhouette score at this point.

Further details on clustering analysis, its interpretation, and implication for policymakers are provided in the subsequent sections.

## 4. Results

Both K-Means and hierarchical clustering were conducted on the processed and scaled data. The results from K-Means clustering is summarized in Table 3. In this table, for each cluster formed as an output of the K-Means algorithm, the mean of each variable used in this study is calculated and summarized, and the values for the same variables and across clusters can be compared. As the table illustrates, for some variables such as SO-VH and HE-MV (except Cluster 4), there are no significant differences across clusters, and, no matter the type of cluster that a certain number of counties are part of across the United States, the mean for these variables do not vary substantially. On the other hand, there are variables such as PO-RE, HE-WI, SO-HE, DE-BP, and DE-HP that differ significantly across clusters, which illustrates the need for further evaluation and analysis of how different the targeted health campaigns should be when addressing these groups of counties within different clusters.

**Figure 3:**
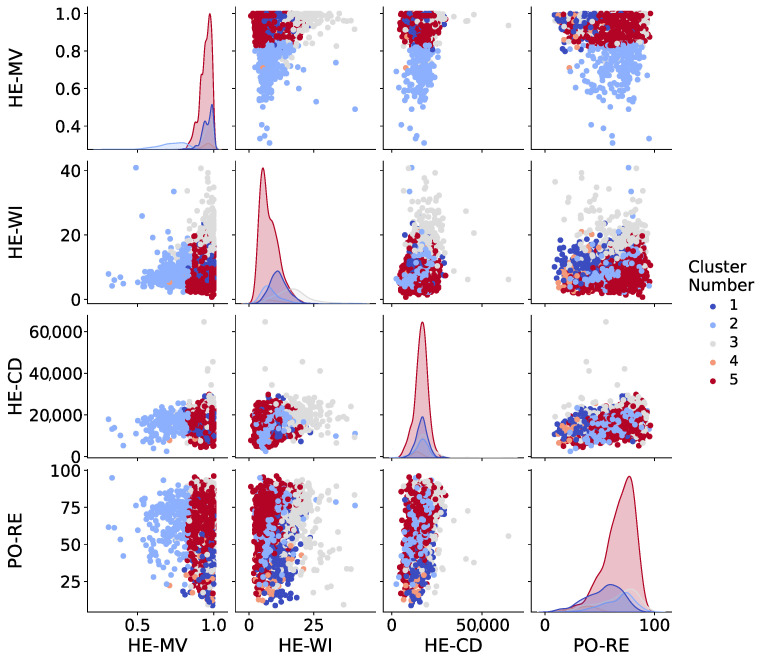
Pairwise plot of key variables colored by K-Means cluster labels. This plot illustrates the distribution and relationships of political preference and health variables across the identified clusters. Each point represents a county, colored according to its cluster assignment. The diagonal elements show the distribution of each variable within clusters. **Variable labels: HE-CD**: case rate December 2021 (cumulative COVID-19 death cases per 100,000 people), **PO-RE**: voting Republican (2020 election) (%), **HE-WI**: without insurance (%), **HE-MV**: measles, mumps, and rubella (MMR) vaccination coverage (0–1).

Pairwise association analyses were also performed to better interpret relationships among variables against one another. Figure 3 illustrates the pairwise relationship among main healthcare-related variables and political preference, where counties are separated by different colors corresponding to their cluster numbers. A more detailed visual is provided in Appendix A.2. As can be observed, some clusters such as Clusters 2 and 5 are distinctly separated from one another in most subplots. Additional information regarding the interpretation and insights is provided in the Section 5.

**Figure 4:**
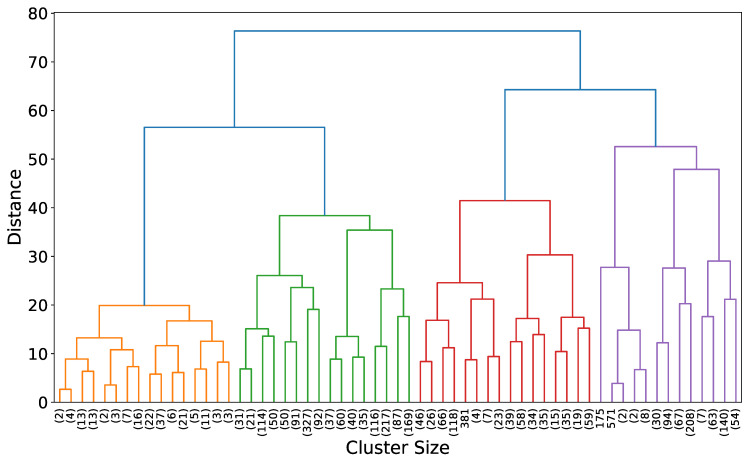
Dendrogram of hierarchical clustering using Ward’s method. This plot visualizes the hierarchical relationships between counties based on key socio-economic and health variables. The height of each branch indicates the dissimilarity between clusters, with lower branches representing more similar clusters. The vertical axis represents the Euclidean distance, which quantifies the dissimilarity between clusters. The colors represent the five distinct clusters identified. To enhance visibility, the line thickness has been increased, and the color palette has been adjusted for clarity.

Additionally, the resulting dendrogram from the hierarchical clustering in Figure 4 illustrates common socio-economic profiles and vaccination behaviors. Figure 4 shows the dendrogram resulting from hierarchical clustering using Ward’s method [45]. This visualization provides insights into the hierarchical relationships and similarities between counties based on the selected socio-economic and health variables. The dendrogram’s branches represent clusters of counties, with the height of each branch indicating the level of dissimilarity between them. The lower branches correspond to more similar clusters, while higher branches indicate greater dissimilarity. The colors differentiate the five distinct clusters identified through the hierarchical clustering process. This hierarchical structure helps to understand the nested grouping of counties, revealing how smaller, more similar clusters combine to form larger, more diverse groups. By examining the dendrogram, one can infer the relative similarity of counties within and between clusters, aiding in the interpretation of socio-economic and health disparities across regions.

In hierarchical clustering, the dendrogram provides a visual representation of the clustering process, illustrating how data points (in this study, counties) are grouped based on their similarities. Each branch in the dendrogram indicates a cluster, with the length of the branch representing the dissimilarity between clusters. The topmost branches in the dendrogram, often shown in a distinct color (such as blue in our case), represent the broadest groupings of data points. These top branches merge clusters at a higher hierarchical level before reaching the specified number of final clusters.

In our analysis, we specified five clusters, and these are visualized by the colored branches (orange, green, red, purple, and blue) in the dendrogram. The different colors help distinguish the distinct clusters formed at the specified level. The top blue branches represent the initial broad groupings, which then split into more refined clusters as we move down the hierarchy. This hierarchical structure allows us to see both the broad and detailed relationships between counties based on their socio-economic and health-related characteristics, providing a comprehensive view of the clustering process.

Following the clustering analysis using K-Means and hierarchical clustering, certain metrics were utilized to evaluate the performance of each model. Additional information regarding the metrics used, and the results of the evaluation are provided in the Appendix A.3 section, which provides the rationale for the conclusion to proceed with K-Means clustering results as a superior model for our analysis. Therefore, in all the following illustrations and analyses, the K-Means clustering results were leveraged.

**Figure 5:**
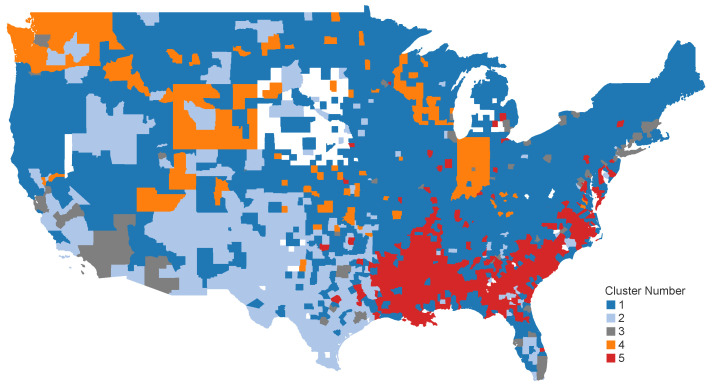
Geospatial distribution of K-Means clusters across the contiguous United States. This map visualizes the geographical locations of counties grouped by their cluster assignments. Each color represents a different cluster, highlighting regional patterns and socio-economic disparities in vaccine hesitancy and other health-related factors.

We, additionally, were interested in identifying the geospatial patterns across the United States based on the clusters formed. Upon displaying the countries across five clusters on the U.S. map, we obtained intriguing results. Figure 5 illustrates the geospatial distribution of counties across the contiguous United States, categorized by their K-Means cluster assignments. Each color on the map corresponds to a different cluster, allowing for a visual comparison of regional patterns in vaccine hesitancy and other socio-economic and health-related variables.

The map reveals distinct regional trends in cluster distribution. For instance, Cluster 5, predominantly located in the southern United States, exhibits higher vaccine hesitancy rates, lower socio-economic status, and higher percentages of minority populations. This suggests that public health interventions in these areas need to address specific socio-economic barriers and cultural factors to improve vaccination rates and health outcomes.

Conversely, clusters in the Northeast and West Coast regions tend to have higher socio-economic status, lower vaccine hesitancy, and better overall health outcomes. These regions may benefit from continued public health education and targeted efforts to maintain high vaccination rates. The geospatial distribution underscores the importance of tailoring public health strategies to regional contexts, considering the unique socio-economic and demographic characteristics that influence health behaviors and outcomes.

**Figure 6:**
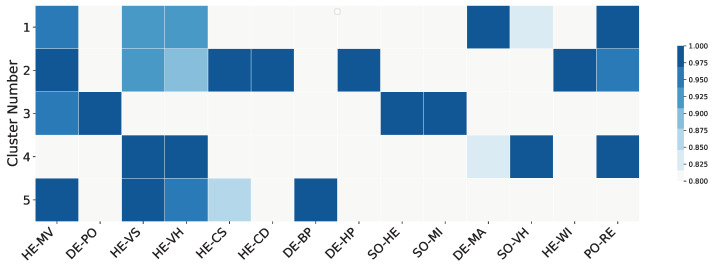
Heatmap of scaled socio-economic and health variables across K-Means clusters. The heatmap visualizes the relative presence of each variable within the identified clusters, with darker shades of blue indicating higher values. This allows for a clear comparison of socio-economic and health characteristics among the clusters. **Variable labels: DE-PO**: population 2020, **HE-VS**: vaccine hesitancy September 2021, **HE-VH**: vaccine hesitancy Dec 2021, **HE-CS**: case rate September 2021 (cumulative COVID-19 death cases per 100,000 people), **HE-CD**: case rate December 2021 (cumulative COVID-19 death cases per 100,000 people), **DE-BP**: Black population (%), **DE-HP**: Hispanic population (%), **SO-HE**: higher education (%), **SO-MI**: median household income, **DE-MA**: median age, **SO-VH**: vehicles per household, **HE-WI**: without insurance (%), **PO-RE**: Republican (%), **HE-MV**: measles, mumps, and rubella (MMR) vaccination coverage (0–1).

Additionally, the heatmap presented in Figure 6 illustrates the scaled values of key socio-economic and health variables across the clusters identified through K-Means clustering. By scaling the variables between 0 and 1, the heatmap provides a normalized view of the data, enabling a straightforward comparison of relative values within each cluster. In this heatmap, darker shades of blue represent higher values, highlighting areas where certain socio-economic or health characteristics are more pronounced. For instance, clusters with darker shades for MMR vaccination coverage (HE-MV) indicate regions with better immunization rates. Conversely, clusters with darker shades for vaccine hesitancy rates in December 2021 (HE-VH) highlight regions with significant resistance to vaccination. The heatmap effectively captures the heterogeneity in socio-economic and health profiles across different clusters. It reveals how certain clusters might have higher median household incomes, lower percentages of uninsured populations, or different racial compositions, which are critical factors influencing public health outcomes. This visualization aids in identifying target areas for intervention and allows policymakers to tailor their strategies to the unique needs of each cluster, ultimately contributing to more effective public health responses.

### Multiple Regression Analysis Results

The regression analysis was performed to identify the key socio-economic and demographic factors that significantly influence vaccine hesitancy across different clusters. Understanding these relationships can help policymakers and public health officials design targeted interventions to increase vaccination uptake and manage pandemic responses more effectively.

The regression analysis follows Equation (1) and utilized a multiple linear regression model with vaccine hesitancy in December 2021 as the dependent variable. The independent variables are the variables listed in Table 1 with the exception of the September 2021 health-related variables HE-VS and HE-CS due to high correlation with the corresponding December variables, and to avoid multicollinearity in the multiple regression analysis. Each cluster’s data were analyzed separately to account for the unique socio-economic and demographic characteristics within each group. It is important to note that the multiple regression analysis in this study focuses on within-cluster analysis to better evaluate the relationship among variables in each of the groups of counties.

(1) $$\begin{array}{cc} \mathrm{HE-VH} & =\beta_{0}\\ \; & +\beta_{1}\times \mathrm{Health}\;\mathrm{Variables}\;(\mathrm{HE-MV},\;\mathrm{HE-CD},\;\mathrm{HE-WI})\\ \; & +\beta_{2}\times \mathrm{Demographic}\;\mathrm{Variables}\;(\mathrm{DE-PO},\;\mathrm{DE-BP},\;\mathrm{DE-HP},\;\mathrm{DE-MA})\\ \; & +\beta_{3}\times \mathrm{Socio-economic}\;\mathrm{Variables}\;(\mathrm{SO-HE},\;\mathrm{SO-MI},\;\mathrm{SO-VH})\\ \; & +\beta_{4}\times \mathrm{Political}\;\mathrm{Variables}\;(\mathrm{PO-RE})\\ \; & +\epsilon \end{array}$$

The regression results provided in Table 4 indicate that different variables significantly influence vaccine hesitancy in each cluster. For instance, in Cluster 1, the MMR vaccination coverage, COVID-19 death rate, and percentage of Republican voters were significant predictors of vaccine hesitancy. Higher MMR vaccination coverage was associated with lower vaccine hesitancy, while higher COVID-19 death rates and a higher percentage of Republican voters were associated with increased vaccine hesitancy.

In Cluster 2, higher education levels and median household income were significant factors reducing vaccine hesitancy. This cluster also showed that racial composition, specifically the percentage of Black population, had a significant positive association with vaccine hesitancy, indicating higher hesitancy rates among these communities. Cluster 3 highlighted the importance of socio-economic status, with median household income and education levels being significant predictors. Political affiliation, again, played a crucial role, with areas having higher Republican affiliations showing increased vaccine hesitancy.

The findings from the regression analysis underscore the multi-faceted nature of vaccine hesitancy. Socio-economic factors such as education and income, along with demographic characteristics like race and political affiliation, play critical roles in shaping vaccination behaviors. Public health strategies must, therefore, be multi-faceted and tailored to address these diverse factors. For instance, interventions in areas with high Republican affiliation might focus on bipartisan messaging to reduce vaccine hesitancy. In contrast, regions with significant minority populations may benefit from community-specific outreach programs that address cultural and historical factors contributing to hesitancy.

Overall, the regression analysis provides valuable insights into the determinants of vaccine hesitancy across different socio-economic contexts. These findings can inform targeted public health interventions, ensuring that efforts to increase vaccine uptake are both effective and equitable.

## 5. Discussion

The results of our clustering analysis offer several important insights into the socio-economic factors influencing COVID-19 vaccine hesitancy across U.S. counties. By examining the profiles of the identified clusters, we can better understand the complex interplay between vaccination behaviors and various socio-economic variables. This discussion section delves into the implications of our findings, the limitations of the study, and potential directions for future research projects.

### 5.1. Insights and Implications

The analysis reveals that socio-economic factors play a significant role in shaping vaccine hesitancy behaviors. Counties with higher median household incomes, higher education levels, and more diverse racial compositions generally exhibited lower vaccine hesitancy rates. This underscores the importance of socio-economic stability in facilitating vaccine uptake. Public health initiatives targeting low-income and low-education areas could benefit from addressing these socio-economic barriers to improve vaccination rates. The geographic distribution of the clusters across the contiguous United States reveals significant insights into the socio-political and demographic characteristics influencing COVID-19 vaccine hesitancy rates and case rates.

Cluster 5, predominantly located in the U.S. South, corresponds to counties with high vaccine hesitancy rates (approximately 47.81%) and moderate COVID-19 case rates (approximately 16,424 for December 2021). These counties also exhibit higher Black population percentages (approximately 35.99%) and moderate socio-economic status. This region’s vaccination uptake could be influenced by public health efforts targeted at minority populations and the presence of robust community health programs.

Cluster 3 is mainly found in large urban centers such as Boston, NYC, Miami, Houston, SFO, and LAX. Counties in this cluster generally show low vaccine hesitancy rates (approximately 24.43%) and moderate COVID-19 case rates (approximately 13,584 for December 2021). The socio-economic status in these areas is higher, with significant percentages of higher education (approximately 66.49%) and median household incomes. The lower Republican affiliation in these counties (approximately 34.83%) might suggest a correlation between political ideology and lower vaccine hesitancy, which has been observed in various studies. The presence of higher Black populations (approximately 15.10%) in these areas also indicates that socio-economic disparities and access to healthcare could play a significant role in vaccination and case rates.

Clusters 1 and 2 are primarily situated in traditionally Democrat and liberal areas, including parts of the Northeast, West Coast, and some urban centers. Cluster 1, with moderate vaccine hesitancy rates (approximately 45.75%) and case rates, is characterized by balanced racial compositions and moderate socio-economic status. Cluster 2, also found in Democrat regions, shows lower vaccine hesitancy rates (approximately 44.96%) and case rates but stands out with a higher socio-economic status, including significant percentages of higher education (approximately 49.13%) and median household incomes.

These clusters underscore the importance of socio-economic factors and political climates in shaping public health outcomes. The higher socio-economic status in these areas likely contributes to better access to healthcare resources and public health information, influencing vaccination behaviors and outcomes.

Overall, the spatial analysis of the clusters highlights the complex interplay between political affiliation, socio-economic status, and public health responses to the COVID-19 pandemic. The distinct regional patterns suggest that targeted public health strategies considering local socio-political and economic contexts are crucial for improving vaccination uptake and managing COVID-19 case rates effectively.

Our analysis highlights the significant role that socio-economic factors play in shaping vaccine hesitancy. The identified clusters reveal distinct patterns, with higher socio-economic status generally correlating with lower vaccine hesitancy. For instance, counties in Cluster 2, characterized by higher education levels and median household incomes, exhibit lower vaccine hesitancy rates. Conversely, Cluster 5, with the lowest socio-economic status, shows the highest vaccine hesitancy rates. This suggests that improving socio-economic conditions could be a key strategy in reducing vaccine hesitancy.

The geographical distribution of clusters also underscores regional disparities. Clusters with higher vaccine hesitancy rates are often found in areas with higher proportions of Republican voters, indicating that political affiliation significantly influences vaccination behaviors. Public health campaigns must consider these political and regional differences to design more effective interventions.

#### 5.1.1. Racial and Ethnic Disparities

Our study highlights substantial racial and ethnic disparities in vaccine hesitancy rates. Clusters with higher percentages of Black and Hispanic populations showed varying vaccination behaviors. For example, Cluster 3, characterized by a significant Black population (15.10%) and lower socio-economic status, exhibited the lowest vaccine hesitancy rates (24.43%) but also faced moderate COVID-19 case rates. This indicates that, while vaccine hesitancy might be lower in some demographics, other factors such as healthcare access and underlying health conditions may still pose challenges. Tailored public health messages and interventions that address specific community needs are essential.

#### 5.1.2. Political Affiliation

Political affiliation emerged as a crucial factor influencing vaccine hesitancy rates. Counties with a higher percentage of Republican voters tended to have higher vaccine hesitancy rates and higher COVID-19 case rates. For instance, Cluster 1 and Cluster 2, with significant Republican affiliations (68.13% and 66.45%, respectively), exhibited moderate to high vaccine hesitancy rates (45.75% and 44.96%). This finding aligns with previous research indicating that political beliefs significantly impact health behaviors and attitudes towards vaccination. Public health campaigns need to consider these political disparities and strive to develop bipartisan messaging that can resonate across different political landscapes.

#### 5.1.3. Clusters and Public Health Interventions

The distinct profiles of the identified clusters provide valuable information for designing targeted public health interventions. For instance, Cluster 1, which exhibits moderate vaccine hesitancy rates (45.75%) and a relatively high socio-economic status, can serve as a model for best practices in addressing vaccine hesitancy through educational campaigns and improving access to vaccines. In contrast, Cluster 5, with the highest vaccine hesitancy rates (47.05%) and lowest socio-economic status, requires urgent and targeted interventions. Public health strategies in these areas should focus on addressing socio-economic barriers and enhancing community engagement to improve vaccine uptake.

Overall, the analysis emphasizes the need for tailored public health strategies that consider the socio-economic and political context of different regions. By understanding the unique characteristics of each cluster, policymakers can implement more effective interventions to reduce vaccine hesitancy and manage public health responses more efficiently.

#### 5.1.4. Final Thoughts

A U.S.-national cluster analysis is necessary in the post-pandemic era to identify regions that are at risk for low vaccine uptake in future vaccination campaigns. Understanding the socio-economic and demographic factors that contributed to vaccine hesitancy during the COVID-19 pandemic can help public health officials design targeted interventions for future health crises. By recognizing these patterns, we can develop strategies to address vaccine hesitancy more effectively and ensure higher vaccination rates across the country.

The findings from our study provide valuable insights into the socio-economic and demographic factors that influence vaccine hesitancy. These insights can be used to inform public health policies and vaccination campaigns in the post-pandemic era. For example, areas identified as high-risk for vaccine hesitancy can be targeted with tailored public health messages and resources to improve vaccine uptake. Additionally, understanding the impact of political affiliation on vaccination behaviors can help in developing bipartisan public health campaigns that resonate across different political landscapes.

Additionally, the findings from this work can be generalized to other vaccination situations by applying the same clustering techniques and variable selection criteria. The socio-economic and demographic factors that influence vaccine hesitancy are likely to be relevant for other vaccines as well. By using a similar approach, public health officials can identify regions at risk for low uptake of other vaccines and design targeted interventions to improve vaccination rates. This generalizability makes our study a valuable tool for addressing vaccine hesitancy beyond the COVID-19 pandemic.

### 5.2. Limitations

#### 5.2.1. Data Limitations

While the study leverages comprehensive data from multiple sources, there are inherent limitations. The accuracy and completeness of the data can vary, and missing values were handled by removing incomplete rows, which could introduce bias. Additionally, the data are static and reflect a specific time period; therefore, temporal changes in vaccine hesitancy rates and socio-economic factors are not captured. Future research could benefit from longitudinal data to observe how these factors evolve over time.

#### 5.2.2. Generalizability

The findings are specific to the U.S. context and may not be directly applicable to other countries with different socio-economic and political landscapes. Comparative studies across countries could provide broader insights into global vaccination behaviors. Understanding how different health policies and cultural contexts affect vaccine hesitancy would help generalize the findings and inform international public health strategies.

## 6. Conclusions

This study underscores the critical role of socio-economic factors in influencing COVID-19 vaccine hesitancy across the U.S. counties. The hierarchical clustering analysis reveals distinct clusters with unique socio-economic profiles and vaccination behaviors, providing invaluable insights for targeted public health interventions. Addressing the socio-economic barriers and disparities identified in this study is essential for improving vaccine uptake and managing pandemic responses more effectively. Future research should continue to explore these complex relationships, incorporating dynamic, interventional, and qualitative approaches to build a comprehensive understanding of vaccination behaviors and public health outcomes.

### Future Research

Future research could incorporate a temporal dimension to examine how vaccine hesitancy rates and socio-economic factors evolve over time. This would provide a more dynamic understanding of vaccination behaviors and the impact of changing socio-economic conditions. Moreover, studies evaluating the effectiveness of specific public health interventions in different clusters would be invaluable. By testing and comparing various strategies, researchers can identify the most effective approaches to increase vaccination rates in different socio-economic contexts.

Expanding the range of socio-economic variables to include factors such as employment status, healthcare infrastructure, and social capital could provide deeper insights into the determinants of vaccination behaviors. Finally, integrating qualitative research methods, such as interviews and focus groups, can enrich the understanding of vaccine hesitancy and acceptance. These methods can capture the nuanced perspectives and experiences of individuals within different clusters, offering a more comprehensive view of the factors influencing vaccine behaviors.

## Figures and Tables

**Table 2:** Uses/Domains of application of computational methods employed.

Method	Use/Domain of Application
**Cumulative Explained Variance**	Determining the optimal number of principal components to retain for capturing the majority of the variance in the dataset.
**Silhouette Score**	Evaluating the separation distance between resulting clusters to determine the optimal number of clusters.
**Correlation Analysis**	Understanding relationships between different socio-economic, demographic, and health variables.
**K-Means Clustering**	Identifying distinct groups of counties with similar socio-economic and demographic profiles.
**Hierarchical Clustering**	Understanding the hierarchical relationships and similarities between counties.
**Davies–Bouldin Index**	Measuring the average similarity ratio of each cluster with its most similar cluster.
**Calinski–Harabasz Index**	Assessing the ratio of the sum of between-clusters dispersion and within-cluster dispersion to evaluate clustering performance.
**Multiple Regression Analysis**	Identifying key socio-economic and demographic factors that significantly influence vaccine hesitancy across different clusters.
**Density Diagrams (Plots)**	Visualizing the distribution of key socio-economic and health variables across different clusters for comparative analysis.

**Table 3:** Mean values of variables for each cluster. **Variable labels: DE-PO**: population 2020, **HE-VS**: vaccine hesitancy September 2021, **HE-VH**: vaccine hesitancy December 2021, **HE-CS**: case rate September 2021 (cumulative COVID-19 death cases per 100,000 people), **HE-CD**: case rate December 2021 (cumulative COVID-19 death cases per 100,000 people), **DE-BP**: Black population (%), **DE-HP**: Hispanic population (%), **SO-HE**: higher education (%), **SO-MI**: median household income, **DE-MA**: median age, **SO-VH**: vehicles per household, **HE-WI**: without insurance (%), **PO-RE**: Republican (%), **HE-MV**: measles, mumps, and rubella (MMR) vaccination coverage (0–1).

Variable	Cluster 1	Cluster 2	Cluster 3	Cluster 4	Cluster 5
**HE-MV**	0.95	0.95	0.95	0.72	0.96
**DE-PO**	64,038.23	78,758.02	1,578,193.00	60,299.54	87,438.19
**HE-VS**	51.28	51.38	33.15	52.87	52.31
**HE-VH**	45.75	44.96	24.43	47.81	47.05
**HE-CS**	12,387.94	15,179.48	11,626.60	12,064.21	14,734.83
**HE-CD**	16,352.73	18,244.01	13,584.15	16,424.87	16,748.07
**DE-BP**	3.58	4.48	15.10	1.85	35.99
**DE-HP**	5.71	35.22	22.77	6.20	5.51
**SO-HE**	54.61	49.13	66.49	55.48	49.04
**SO-MI**	57,193.94	53,114.07	77,931.01	58,473.19	46,155.63
**DE-MA**	43.05	36.59	37.51	41.89	39.15
**SO-VH**	2.02	1.96	1.67	2.10	1.79
**HE-WI**	7.83	16.61	8.37	8.65	11.44
**PO-RE**	68.13	66.45	34.83	67.11	53.79

**Table 4:** Regression analysis of socio-economic and demographic variables on vaccine hesitancy (December 2021). **Variable labels: DE-PO**: population 2020, **HE-VS**: vaccine hesitancy September 2021, **HE-VH**: vaccine hesitancy December 2021, **HE-CS**: case rate September 2021 (cumulative COVID-19 death cases per 100,000 people), **HE-CD**: case rate December 2021 (cumulative COVID-19 death cases per 100,000 people), **DE-BP**: Black population (%), **DE-HP**: Hispanic population (%), **SO-HE**: higher education (%), **SO-MI**: median household income, **DE-MA**: median age, **SO-VH**: vehicles per household, **HE-WI**: without insurance (%), **PO-RE**: Republican (%), **HE-MV**: measles, mumps, and rubella (MMR) vaccination coverage (0–1).

	Dependent Variable: Vaccine Hesitancy (December 2021)				
	**Cluster 1**	**Cluster 2**	**Cluster 3**	**Cluster 4**	**Cluster 5**
**Health**					
HE.CD	−**0.250** *** (0.044)	−**0.316** *** (0.069)	−0.309 (0.256)	−0.196 (0.128)	0.033 (0.094)
HE.WI	0.036 (0.027)	−**0.121** *** (0.043)	−0.128 (0.151)	**0.205** *** (0.057)	**0.276** *** (0.057)
HE.MV	−**0.128** *** (0.039)	−**0.175** ** (0.089)	−0.190 (0.140)	−**0.135** *** (0.043)	−**0.279** *** (0.083)
**Demographic**					
DE.PO	−**0.480** * (0.251)	−0.374 (0.424)	0.090 (0.077)	0.507 (0.638)	−0.033 (0.396)
DE.BP	**0.205** *** (0.039)	**0.437** *** (0.099)	**0.370** *** (0.133)	0.012 (0.135)	0.041 (0.051)
DE.HP	−0.024 (0.038)	−**0.084** *** (0.031)	−0.028 (0.139)	−**0.348** *** (0.105)	−0.083 (0.126)
DE.MA	−**0.257** *** (0.021)	−**0.243** *** (0.056)	−**0.631** *** (0.179)	−**0.145** ** (0.056)	−**0.302** *** (0.052)
**Socio-economic**					
SO.HE	−**0.071** *** (0.025)	−**0.183** *** (0.069)	0.027 (0.204)	−**0.184** *** (0.060)	−**0.222** *** (0.061)
SO.MI	−**0.309** *** (0.029)	−**0.139** * (0.071)	−**0.240** ** (0.104)	−**0.194** ** (0.084)	−**0.188** ** (0.078)
SO.VH	**0.325** *** (0.036)	0.007 (0.101)	**0.235** ** (0.111)	**0.247** ** (0.112)	**0.304** *** (0.084)
**Political**					
PO.RE	**0.590** *** (0.023)	**0.665** *** (0.034)	**0.472** *** (0.150)	**0.503** *** (0.064)	**0.289** *** (0.053)
Constant	**0.317** *** (0.047)	**0.580** *** (0.120)	**0.497** ** (0.197)	**0.372** *** (0.076)	**0.648** *** (0.102)
Observations	1795	377	79	242	474
R-squared	0.669	0.681	0.630	0.652	0.500
Adj. R-squared	0.667	0.671	0.569	0.635	0.488

* *p* < 0.01; ** *p* < 0.05; *** *p* < 0.01.

**Figure A1:**
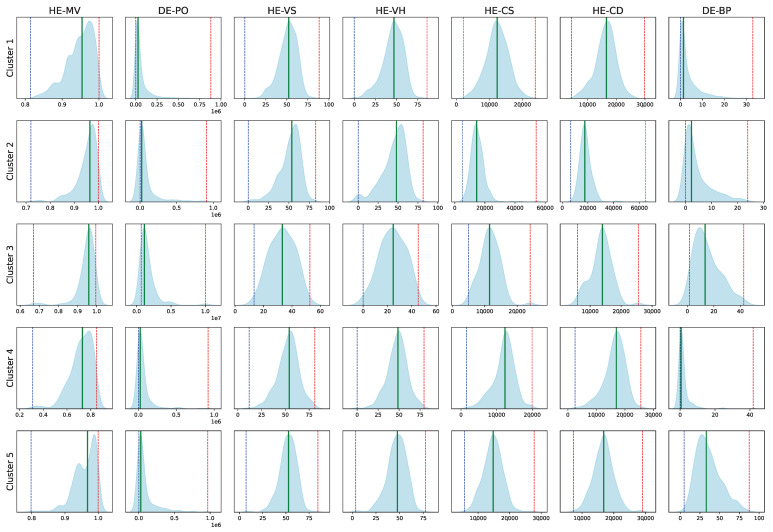
Density plot (part 1) of selected socio-economic and health variables across different clusters. Variables include **HE-MV: measles, mumps, and rubella (MMR) vaccination coverage (0–1)**, **DE-PO: population 2020**, **HE-VS: vaccine hesitancy September 2021 (%)**, **HE-VH: vaccine hesitancy December 2021 (%)**, **HE-CS: cumulative COVID-19 case rates per 100,000 people as of September 2021**, **HE-CD: cumulative COVID-19 case rates per 100,000 people as of December 2021**, and **DE-BP: Black population (%)**. Each subplot shows the distribution of a specific variable within each cluster, providing a comparative analysis of how these variables vary among different clusters. The vertical lines indicate the means for each cluster, with colors corresponding to the different clusters.

**Figure A2:**
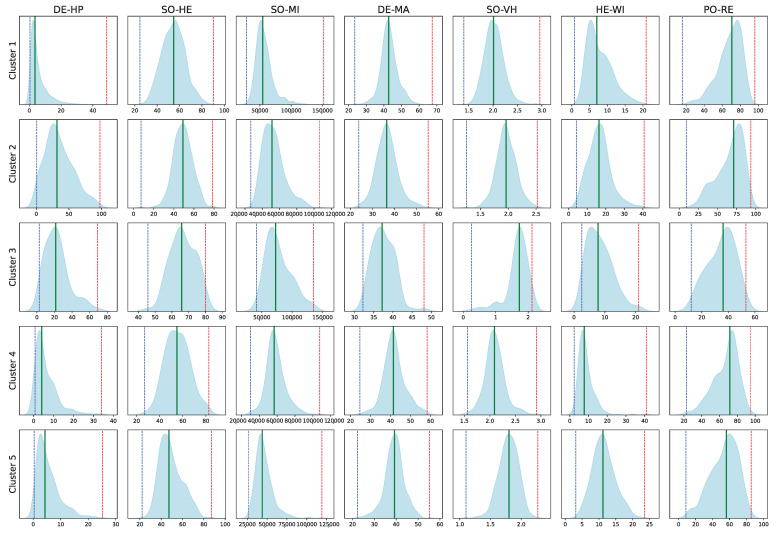
Density plot (part 2) of selected socio-economic and health variables across different clusters. Variables include **DE-HP: Hispanic population (%)**, **SO-HE: higher education (%)**, **SO-MI: median household income**, **DE-MA: median age**, **SO-VH: vehicles per household**, **HE-WI: without insurance (%)**, and **PO-RE: Republican (%)**. Each subplot shows the distribution of a specific variable within each cluster, providing a comparative analysis of how these variables vary among different clusters. The vertical lines indicate the means for each cluster, with colors corresponding to the different clusters.

**Figure A3:**
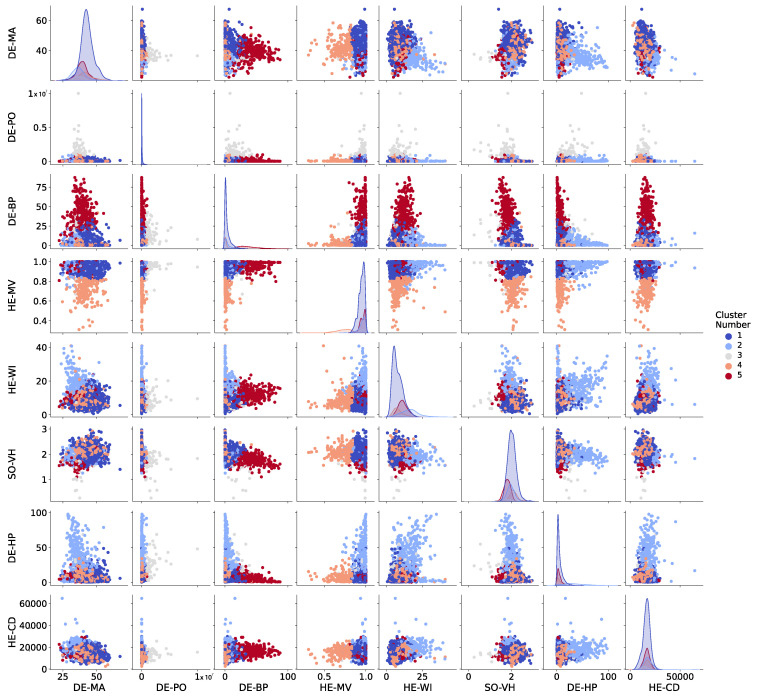
Pairwise plot of key variables colored by K-Means cluster labels. This plot illustrates the distribution and relationships of key socio-economic and health variables across the identified clusters. Each point represents a county, colored according to its cluster assignment. The diagonal elements show the distribution of each variable within clusters. **Variable labels: HE-CD**: case rate Dec 2021 (cumulative COVID-19 death cases per 100,000 people), **DE-HP**: Hispanic population (%), **DE-MA**: median age, **SO-VH**: vehicles per household, **HE-WI**: without insurance (%), **HE-MV**: measles, mumps, and rubella (MMR) vaccination coverage (0–1).

## Data Availability

The data and code for this study’s analysis can be found in the following GitHub repository: https://github.com/mprtrmrtz/covid-clustering (accessed on 18 July 2024).

## Abbreviations

The following abbreviations are used in this manuscript:

PCA	Principal Component Analysis
ML	Machine Learning
ERS	Economic Research Service
USDA	United States Department of Agriculture
JHU	Johns Hopkins University
JHU CSSE	Johns Hopkins University Center for Systems Science and Engineering
AIC	Akaike Information Criterion
BIC	Bayesian Information Criterion
K-Means	K-Means Clustering
CH Index	Calinski–Harabasz Index
DB Index	Davies–Bouldin Index
VH	Vaccine Hesitancy
COVID-19	Coronavirus Disease 2019
CDC	Centers for Disease Control and Prevention
WHO	World Health Organization
HHS	Department of Health and Human Services
VAX	Vaccine Administration
MLR	Multiple Linear Regression
SVD	Singular Value Decomposition
RMSE	Root Mean Square Error
MAE	Mean Absolute Error
GIS	Geographic Information System
FIPS	Federal Information Processing Standards
MMR	Measles, Mumps, and Rubella

## Appendix A

### Appendix A.1. Density Plots

The density plots presented in Figure A1 and Figure A2 provide a detailed visualization of the distribution of key socio-economic and health variables across the identified clusters. Each subplot represents the density of a specific variable within each cluster, allowing for a comparative analysis of how these variables vary among different clusters. In the first set of density plots (Figure A1), we observe the distributions of variables such as MMR vaccination coverage (HE-MV), population 2020 (DE-PO), and vaccine hesitancy rates in September and December 2021 (HE-VS and HE-VH). These plots reveal distinct patterns, indicating significant variability in these variables across clusters. For instance, some clusters show higher density peaks for MMR vaccination coverage, suggesting better immunization rates in those regions, while others demonstrate higher vaccine hesitancy rates, indicating a need for targeted public health interventions. The second set of density plots (Figure A2) continues this analysis with additional variables, including case rates in September and December 2021 (HE-CS and HE-CD), Black population percentage (DE-BP), and Hispanic population percentage (DE-HP). These plots further highlight the socio-economic and racial disparities among the clusters. For example, clusters with higher case rates might correlate with lower vaccination coverage or higher vaccine hesitancy, underscoring the complex interplay between health outcomes and socio-economic factors.

Overall, the density plots provide a comprehensive overview of the heterogeneity in socio-economic and health profiles across different clusters. They serve as a crucial tool for identifying target areas for public health interventions, enabling policymakers to tailor strategies to the unique needs of each cluster.

### Appendix A.2. Pairwise Plot

Figure A3 presents a pairwise plot of key socio-economic and health variables, with points colored by their K-Means cluster assignments. This plot offers a comprehensive view of how different variables distribute and interact across the clusters. Each diagonal subplot displays the distribution of a specific variable within the clusters, while the off-diagonal subplots show the bivariate relationships between variables.

The pairwise plot reveals clear distinctions between clusters, highlighting the heterogeneity in vaccination hesitancy, socio-economic status, and COVID-19 case rates among counties. For instance, clusters with higher vaccine hesitancy rates tend to have lower median household incomes and higher percentages of the population without insurance. These insights underscore the complex interplay between socio-economic factors and health outcomes, suggesting targeted interventions could be tailored for specific clusters to address underlying disparities.

### Appendix A.3. Clustering Performance Metrics

In order to further validate the results, we computed the silhouette score [46], Davies–Bouldin Index [47], and Calinski–Harabasz Index [48] for each clustering task between K-Means and hierarchical clustering. The silhouette score indicates how similar an object is to its own cluster compared to other clusters, with higher values indicating better-defined clusters. The Davies–Bouldin Index measures the average similarity ratio of each cluster with its most similar cluster, where lower values represent better clustering. The Calinski–Harabasz Index assesses the ratio of the sum of between-clusters dispersion and within-cluster dispersion, with higher values indicating better clustering. These metrics were obtained for both K-Means and hierarchical clustering and are summarized in Table A1. The obtained scores suggest that the K-Means clustering yielded superior results compared to hierarchical clustering, as indicated by higher silhouette scores and lower Davies–Bouldin Indices. Therefore, for the remainder of the analysis, the clusters formed by the K-Means method (five clusters) were leveraged.

**Table A1:** Comparison of clustering performance metrics for hierarchical and K-Means clustering with five clusters. The table displays the silhouette score, Davies–Bouldin Index, and Calinski–Harabasz Index for both clustering methods.

Metric	K-Means (5 Clusters)	Hierarchical
Silhouette Score	0.249	0.181
Davies-Bouldin Index	1.327	1.497
Calinski-Harabasz Index	492.180	373.873
